# Trust Your Gut: The Association of Gut Microbiota and Liver Disease

**DOI:** 10.3390/microorganisms10051045

**Published:** 2022-05-18

**Authors:** Ridda Manzoor, Weshah Ahmed, Nariman Afify, Mashal Memon, Maryam Yasin, Hamda Memon, Mohammad Rustom, Mohannad Al Akeel, Noora Alhajri

**Affiliations:** 1 College of Medicine and Health Sciences, Khalifa University, Abu Dhabi P.O. Box 127788, United Arab Emirates; 100052897@ku.ac.ae (R.M.); 100043004@ku.ac.ae (W.A.); 100049875@ku.ac.ae (N.A.); 100049880@ku.ac.ae (M.M.); 100052896@ku.ac.ae (M.Y.); 100058242@ku.ac.ae (H.M.); 100058284@ku.ac.ae (M.R.); 2 Division of Family Medicine, Department of Health, Abu Dhabi P.O. Box 5674, United Arab Emirates; mohanadalakeel@gmail.com; 3 Department of Medicine, Sheikh Shakhbout Medical City (SSMC), Abu Dhabi P.O. Box 11001, United Arab Emirates

**Keywords:** dysbiosis, *Firmicutes*, *Bacteroidetes*, liver disease, NALFD, ALD, liver cirrhosis, hepatocellular carcinoma, autoimmune hepatitis

## Abstract

The gut microbiota composition is important for nutrient metabolism, mucosal barrier function, immunomodulation, and defense against pathogens. Alterations in the gut microbiome can disturb the gut ecosystem. These changes may lead to the loss of beneficial bacteria or an increase in potentially pathogenic bacteria. Furthermore, these have been shown to contribute to the pathophysiology of gastrointestinal and extra-intestinal diseases. Pathologies of the liver, such as non-alcoholic liver disease, alcoholic liver disease, cirrhosis, hepatocellular carcinoma, autoimmune hepatitis, viral hepatitis, and primary sclerosing cholangitis have all been linked to changes in the gut microbiome composition. There is substantial evidence that links gut dysbiosis to the progression and complications of these pathologies. This review article aimed to describe the changes seen in the gut microbiome in liver diseases and the association between gut dysbiosis and liver disease, and finally, explore treatment options that may improve gut dysbiosis in patients with liver disease.

## 1. Introduction

Each individual has a unique gut microbiota profile that regulates many key functions. The gut microbiota is composed of non-pathogenic bacteria, eukaryotic microorganisms, viruses, parasites, and archaea that colonize the gastrointestinal tract [1]. *Bacteroidetes* and *Firmicutes* constitute 90% of the bacteria in the human digestive tract [2]. 

Over the last decade, there has been exponential growth in the literature that has accumulated in describing the gut microbiota and its relationship to both health and disease [3,4]. The collective genomes of these bacteria encode more than 150-fold the number of expressive genes than that encoded by the human genome. The gut microbiota encodes over three million genes that produce thousands of beneficial products, whereas the human genome consists of approximately 23,000 genes [5]. These products, together with host bacteria, are responsible for preserving homeostasis and are key regulators of digestion, metabolism, absorption of nutrients, health, and immunity. A disruption of the symbiotic relationship between the microbiota and the host, or dysbiosis, has been associated with several diseases, including a wide range of liver pathologies. The term dysbiosis can be defined as the disturbance in quantity, variety, and/or location of microorganisms. This can result in the reduction in microbial diversity, which can lead to a disturbance in the balance of the *Firmicutes*/*Bacteroidetes* ratio, and an increase in symbiotic bacteria that become pathogenic under certain conditions [6].

There has been a growing number of evidence that demonstrates a bidirectional relationship between the gut microbiota and the liver and many interlinked factors that include: genetics, the environment, and diet, which play a role in contributing to dysbiosis [7,8,9,10]. The aim of this review was to outline how microbiota and the liver interact with each other. We focused on the general role of the microbiota as well as the role it plays in liver diseases such as nonalcoholic fatty liver disease (NAFLD), nonalcoholic steatohepatitis (NASH), cirrhosis, autoimmune hepatitis (AIH), and hepatocellular carcinoma (HCC) as indicated in the current literature. This review also addressed some current regimens that utilize dysbiosis for treating liver pathologies. 

In this review article, we explored the association between disturbances in the microbial ecosystem and various liver diseases, with a focus on bacterial changes. We searched PubMed and Google Scholar using the following mesh terms: “gut dysbiosis”, “mycobiota disturbance”, “virome disturbance”, “intestinal ecosystem”, “NASH”, “NAFLD”, “liver cirrhosis” “autoimmune hepatitis” “hepatocellular carcinoma”, “primary biliary sclerosis”, and “primary sclerosing cholangitis”. We explored data from various geographical regions including Asia, Europe, and North America and looked at the composition of various bacterial phyla and species.

### 1.1. Role of Gut–Liver Axis in Liver Disease

The term gut–liver axis was created to demonstrate the intimate relationship among the intestine and liver which involves a complex relationship between the gut microbiome, the immune system, and the intestinal barrier [11]. The liver receives 75% of its blood from the intestines via the portal vein. It also provides feedback to the intestines through the secretion of bile, bile acids, and other mediators. 

The interface between the liver and the microbiota is the intestinal epithelium. This structure aids in regulating metabolic functions and selectively permitting the absorption of nutrients while simultaneously acting as a restrictive barrier against any unwanted microbes or microbial products. The selective permeability of the intestinal epithelial barrier is maintained by tight junctions that include E-cadherins, desmosomes, claudins, occludins, and junctional adhesion molecules [12]. In addition, the intestinal barrier is reinforced by mucins, immunoglobulins, immune cells, and commensal bacteria. Despite the highly specialized epithelium and barriers that modulate the transport across the intestinal mucosa, the disruption of the intestinal barrier can lead to increased intestinal permeability, causing translocation of pathogens, bacteria, and inflammatory cytokines into the portal circulation, which can result in gut inflammation and dysbiosis [13,14]. The breakdown of the components of the barrier has been associated with consumption of a high-fat diet, antibiotic use, chronic alcohol abuse, and immune-associated inflammatory disease [7].

The growing knowledge of the pathophysiology of the gut–liver axis has resulted in a significant number of reviews and evidence that can drive the development of diagnostic, prognostic, and therapeutic tools [15]. 

### 1.2. Normal Gut Microbiota Composition

The incredibly complex diversity of the gut microbiota comprises many species of microorganisms that include bacteria, bacterial products, yeast, and viruses [5]. The ability to survey the depth of the gut microbiota has improved due to new high-throughput and sequencing methodologies. There have been 2172 species isolated and thoroughly described taxonomically in human beings [16]. However, the dominant gut microbial phyla are *Firmicutes*, *Bacteroidetes*, *Actinobacteria*, *Proteobacteria*, *Fusobacteria*, and *Verrucomicrobia*, with the two phyla *Firmicutes* and *Bacteroidetes* representing 90% of gut microbiota [5]. 

The human gut microbiota differs taxonomically and functionally in each part of the gastrointestinal tract. After birth, the human intestine is relatively sterile [17]. However, increasing evidence suggests that human intestinal microbiota is present before birth [18]. Maternal microbiota forms the first inoculum after birth; with the initiation of feeding, bacterial colonization is introduced. The microbial diversity increases to form an adult-like microbiota by the end of 3–5 years of life [18]. 

The gut microbiota composition is comparatively stable throughout adult life, but it can be altered due to infection, antibiotic use, surgery, age, sex, diet, lifestyle, genetics, environment, and various pathologies [19]. Each individual has a unique microbiota composition, and thus there is no one healthy composition [5]. Deschasaux et al., demonstrated that individuals who share the same ethnicity were grouped together, which suggests that they share a similar gut microbiota [19]. It is also well-known that patients with compromised immune systems or those with liver or inflammatory bowel diseases (IBDs) have an altered microbiota when compared to healthy individuals [20,21]. As such, it is crucial to have a better grasp of the gut microbiota in normal physiology and pathophysiology as it provides an enhanced understanding of the microbial alterations in individual patients, which can lead to selectively targeted novel interventions. 

**Figure 1:**
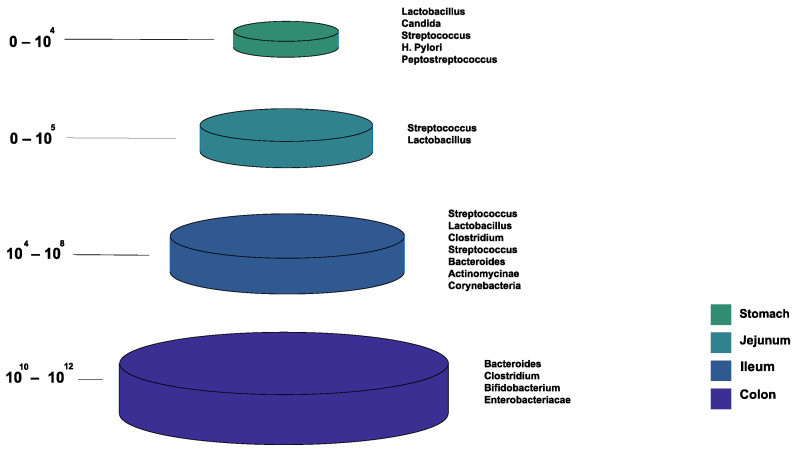
The normal composition of the gut microbiota at different locations of the gastrointestinal tract.

Figure 1 shows the bacterial microbiota composition in various parts of the gut. The gut microbiota is different based on the intestine anatomical regions that vary in terms of physiology, oxygen tension, digestive flow rates (fast in the mouth to the stomach, and slower afterward), and pH [22]. For example, the small intestine has short transit times (3–5 h), while the large intestine is characterized by slower flow rates and neutral pH, accommodating its large microbial community. The total microbiota load in the intestine is about 10^13^–10^14^ microorganisms. We can see a quantitative increase in the gradient as we go down the gut, with a predominance of anaerobic bacteria [17,22]. 

## 2. Gut Microbiota: Link with Non-Alcoholic Liver Disease

### 2.1. Epidemiology, Clinical Manifestations, and Pathophysiology

NAFLD is the most common cause of chronic liver disease worldwide. NAFLD is the deposition of more than 5% of the liver’s weight in the absence of chronic liver disease and significant alcohol intake; this process is known as hepatic steatosis [23,24]. Hepatic steatosis in NAFLD can progress to nonalcoholic fatty liver and NASH. On histology, NASH is defined as evidence of hepatocellular injury and inflammation that can result in fibrosis, which can later progress to liver cirrhosis [23,25]. The prevalence of NAFLD is 25.24% globally. Furthermore, NAFLD is most widespread in the Middle East and South America and least prevalent in Africa [26]. The prevalence of NAFLD in the United States was reported to be 24% in older young adults [27], while the overall prevalence of NASH in the United States is 3% to 6% [28]. The occurrence of HCC caused by NAFLD is around 0.44 in 1000 person-years [26]. Age, sex, type 2 diabetes, metabolic syndrome, ethnicity, genetics, hyperlipidemia, and obesity were connected with an increase in the disease progression of NAFLD [26]. The rates of NAFLD between men and women are the same.

The liver and the gastrointestinal tract have a two-way relationship known as the gut–liver axis. Any change in the composition of this axis contributes to the pathogenesis of NAFLD. This change can be in the form of gut microbiome dysbiosis or gut mucosal barrier damage leading to the development of NAFLD. With the damaged mucosal barrier, bacterial products in NALFD combined with small intestine bacterial overgrowth can play a role in the progression of NAFLD [29,30]. Gut dysbiosis contributes to NAFLD through different mechanisms, classified as inflammatory or metabolic. Inflammatory mechanisms include a decrease in the tight junction protein expression and an increase in ethanol production. Metabolic mechanisms include the alteration in short-chain fatty acids (SCFAs), decrease in fasting-induced adipose factor, alteration in the bile acid profile, and increased conversion of choline to methylamine [29].

A decrease in the tight junction expression leads to gut barrier disruption and increased abundance of PAMPs (e.g., lipopolysaccharides (LPSs)) and DAMPs (e.g., fatty acid) that activate toll-like receptor 4, toll-like receptor 9, and nucleotide-binding domain-associated and leucine-rich repeat pyrin 3 domain, leading to chronic inflammation of the liver. Chronic inflammation of the liver can also be caused by an increase in ethanol production by the gut microbiome. An increase in ethanol production feeds into the inflammatory pathway that causes dysregulation of the endocannabinoid pathway leading to NAFLD [29].

**Figure 2:**
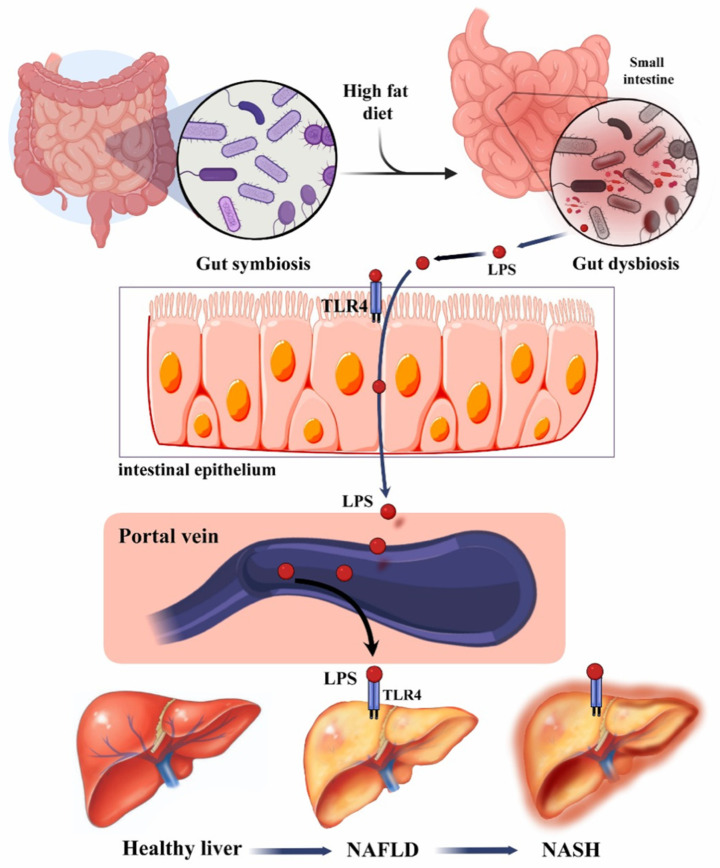
Risk factors and progression of NAFLD to NASH. Adapted with permission from Tokuhara [34].

The role of SCFAs in NAFLD has been a topic of controversy. Some studies have shown that SCFAs increase in NAFLD. SCFAs activate and bind to the G-protein coupled receptor 43; this binding inhibits lipolysis which contributes to fat deposition in adipose tissue in the liver [29,31]. However, Li et al., showed that patients with NAFLD have a reduced abundance of SCFAs that were thought to contribute to liver inflammation [32]. Decrease fasting-induced adipose factor and alteration in bile acid profile contribute to the increase in the liver de novo lipogenesis, which increases the fat mass in the liver. Choline deficiency is associated with a decrease in very low-density lipoprotein (VLDL), which is needed to maintain the liver’s fat content distribution [29,33,34] (Figure 2).

### 2.2. Gut Microbiome Profile in NAFLD

Many studies have shown the relationship between gut dysbiosis and NAFLD and NASH. Generally, *Proteobacteria*, *Actinobacteria*, *Bacteroidetes*, and *Firmicutes* were found to be the main phyla affected by NASH and NAFLD. In patients with NASH or NAFLD, there is an increase in *Proteobacteria* (genus *Escherichia* and other *Enterobacteriaceae* families), *Actinobacteria*, and *Bacteroidetes* (*Bacteroides* and *Prevotella*) [32,35,36,37,38,39]. However, other studies have shown a decrease in *Bacteroidetes* in patients with NASH or NAFLD [37,39,40,41]. Looking at the phylum *Bacteroidetes*, specifically the genus *Prevotella* and *Bacteroides*, there is some controversy. Zhu et al., reported an increased abundance of *Prevotella* and no significant change in *Bacteroides* in obese patients and patients with NAFLD compared to the control [36]. Boursier et al., however, noted that in patients with NASH, there was an increase in *Bacteroides* and a decrease in *Prevotella* [38]. These differences can be attributed to different geographical locations, disease progression, and diets, especially with *Prevotella*, which is associated with high-fiber diets. A number of studies reported increases in *Firmicutes* (*Streptococcus* and *Lachnospiraceae*; genera *Dorea*, *Robinsoniella*, and *Roseburia*) [37,39,40], while other studies showed a decrease in *Firmicutes* (*Ruminococcaceae*, genus *Oscillibacter)* [32,35,36,37,42]. Compared to patients with NAFLD, patients with NASH had an increase in *Firmicutes* and a decrease in *Bacteroidetes*, *Proteobacteria*, and *Actinobacteria* [37,39]. Several studies have reported a decrease in the ratio of *Firmicutes* to *Bacteroidetes* in NAFLD and NASH compared to controls [29,36,42], and others showed an increase in this ratio [39,41]. Other bacterial associations in NAFLD are summarized in Table 1.

**Table 1:** Studies characterizing the composition of the gut microbiota in NAFLD/NASH.

Author Reference	Country	Study Design	Participants	Changes in the Composition of Gut Microbiota in NAFLD	Key Findings
[38]	France	Cross-sectional study	57 NAFLD 35 NASH	↑ *Actinobacteria* ↑ *Bacteroides* ↑ *Ruminococcus* ↓ *Prevotella* No change in *Firmicutes*	↑ *Ruminococcus* was significant in NASH Gut microbiota can be one of the prognostic tools to evaluate NAFLD progression and severity
[40]	Canada	Prospective cross-sectional study	33 NAFLD: 11 simple steatosis 22 NASH 17 healthy controls	↑ *C. Coccoides* in NASH ↓ *Bacteroidetes* in NASH compared to the SS and HCC	The relationship between *Bacteroidetes* and liver disease state was not dependent on the increase in BMI or diet
[41]	United States	Cross-sectional study	44 NAFLD 29 healthy controls	↓ *Bacteroidetes* ↓ *Prevotella* ↓ *Gemmiger* ↓ *Oscillospira*	↓ Bacterial diversity in patients with NAFLD compared to controls contributed to an increase in the rate of inflammation in NAFLD
[35]	United States	Prospective, observational, cross-sectional study	87 NAFLD 37 healthy controls	↑ *Bacteroidetes* ↑ *Proteobacteria* ↓ *Firmicutes*	↓ α-diversity in NAFLD was attributed to the differences in bacterial abundance rather than an increase in specific phyla or genus ↑ Pro-inflammatory bacterial products (LPS) in patients with NAFLD
[36]	United States	Case-control	22 NASH 25 obese 16 healthy controls	↑ *Bacteroides* (*Prevotella*) ↑ *Proteobacteria* (*Escherichia*) ↓ *Firmicutes* ↓ *Actinobacteria*	↑ Abundance of ethanol producing bacteria (*Escherichia*) in patients with NASH contributed to disease progression ↑ Ethanol-producing bacteria (*Escherichia*) was attributed to the use of antibiotics
[39]	Italy	Case-control	61 NASH/NAFL 54 healthy controls	↑ *Actinobacteria* ↑ *Bradyrhizobium* ↑ *Anaerococcus* ↑ *Peptoniphilus* ↑ *Propionibacterium acnes* ↑ *Enterobacteriaceae* (*Escherichia coli*) ↑ *Dorea* ↑ *Ruminococcus* ↓ *Bacteroidetes* ↓ *Oscillospira* ↓ *Rikenellaceae*	↓ Microbial diversity in NASH/NAFL ↓ *Bacteroidaceae* and *Bacteroides* were in NAFL and NASH, while it ↑ in obese patients compared to controls ↑ Ethanol-producing bacteria (*Enterobacteriaceae*) in NAFL/NASH compared to controls
[37]	Canada	Case-control	30 NAFLD 30 healthy controls	↑ *Proteobacteria* ↑ *Firmicutes* ↓ *Bacteroidetes*	Fecal ester volatile organic compounds could influence the microbiome composition of patients with NAFLD in an unfavorable way

BMI, body mass index; HCC, hepatocellular carcinoma; LPS, lipopolysaccharide; NAFL, nonalcoholic fatty liver; NAFLD, nonalcoholic fatty liver disease; NASH, nonalcoholic steatohepatitis; SS, steatohepatitis; ↑, increase; and ↓, decrease.

In general, the differences in the abundance of different phyla between studies can be correlated to age, weight, the severity of NAFLD, ethnicity, geographic location, diet, genetics, and associated comorbidities (e.g., metabolic syndrome). Boursier et al., noted that patients with NAFLD with metabolic syndrome had more severe disease(s) compared to patients with NAFLD without metabolic syndrome. This was because of the increased abundance of *Bacteroides* and *Ruminococcus* [38]. Schwimmer et al., suggested that the difference in bacterial abundance can be attributed to the decrease in α-diversity in NAFLD rather than an increase in specific phlya or genus [35].

Increases in *Escherichia* and other *Enterobacteriaceae* families contribute to intestinal inflammation and liver damage through increasing intestinal permeability and increasing the in vivo production of ethanol [43]. Li et al., reported that *Coprococcus*, *Faecalibacterium*, and *Ruminococcus* produce SCFAs which inhibit some pro-inflammatory markers such as tumor necrosis factor alpha (TNF-α) and monocyte chemotactic protein-1. In NAFLD, there is decreased abundance of these bacteria; hence, there is a decrease in the production of SCFAs contributing to the progression of liver inflammation [32].

## 3. Gut Microbiota: Link with Alcoholic Liver Disease

Alcohol-associated liver disease (ALD) is one of the most common causes of liver disease. It can range from steatosis to steatohepatitis, alcoholic hepatitis, fibrosis, and finally, liver cirrhosis [17]. Alcohol and its metabolites strongly modulate the gut microbiome through its effects on the epithelial and mucosal barrier, as it impairs both the production of antimicrobial peptides and the creation of a pro-inflammatory environment [8,44]. Metagenomic analyses in both humans and alcohol-fed mice show that ALD leads to decreased bacterial diversity. Mutlu et al., analyzed the microbiota composition of colonic biopsies from chronic alcoholics with and without ALD. They demonstrated that those with ALD had a lower abundance of *Bacteroidetes* and *Firmicutes* species and a higher abundance of *Proteobacteria* [45]. The dysbiosis of the microbiota decreases the production of long-chain fatty acids, which are critical for the growth of certain bacteria, such as *Lactobacillus* [8]. 

Chronic alcohol use is associated with increased intestinal inflammation, which involves high levels of TNF-⍺ that are being produced by monocytes and macrophages in the intestinal lamina propria. This leads to the disruption of tight junctions between intestinal cells. This process increases intestinal permeability and allows for the translocation of bacterial products into the systemic circulation and worsening liver inflammation [46]. Inflammation is further aggravated by a loss of protective enzymes important for antibacterial activity. These proteins are the first line of defense against pathogens and help maintain homeostasis of the gut microbiome. Wang et al., discovered that a deficiency of the proteins REG3B and REG3G in mice promoted the development of ethanol-induced steatohepatitis. Loss of these proteins led to increased bacterial translocation to the liver and mesenteric lymph nodes and increased bacterial adhesion to the mucosal layer [47].

## 4. Gut Microbiota: Link with Liver Cirrhosis

### 4.1. Epidemiology, Clinical Manifestations, and Pathophysiology

Liver cirrhosis is the end stage of liver disease and is a leading cause of morbidity and mortality worldwide [48]. In 2017, according to the Global Burden of Disease Study, cirrhosis was the cause of 2.4% of deaths globally. More than 60% of the deaths related to liver disease in 2017 were in men [49]. In the United States, liver cirrhosis is the 10th leading cause of death [47]. The most common causes of cirrhosis are alcohol-related liver disease, NAFLD, and chronic hepatitis B (CHB) and C infection. Risk factors for cirrhosis include alcohol consumption, metabolic syndrome, untreated or chronic viral infection, genetic predisposition to certain diseases such as Wilson’s disease, and autoimmune diseases such as primary biliary cholangitis (PBC) [50].

Long-standing inflammation due to the causes listed above leads to the development of fibrosis, and eventually, cirrhosis [50]. Chronic parenchymal injury causes apoptosis and necrosis of hepatocytes. This activates Kupffer cells, endothelial cells, platelets, and leukocytes. The leukocytes generate cytokines such as TGF beta, IL-1, TGF alpha, PDGF, and EGF, lipid peroxides, and reactive oxygen species. This stimulates the regeneration of nearby hepatocytes, which ultimately leads to nodule formation [51]. Exposure to inflammatory cytokines also activates hepatic stellate cells and initiates fibrosis and deposition of the extracellular matrix at a rate where production is higher than degradation, leading to the loss of healthy liver parenchyma. This results in thickened hepatic septae and collagen cross-linking [52]. Due to the increased fibrosis, the endothelial fenestrations and hepatocyte microvilli are lost, impairing the bidirectional metabolic exchange between the portal venous blood flow and the hepatocytes. This ultimately leads to the development of portal hypertension [52].

Once cirrhosis is present, the patient can remain asymptomatic for years before showing symptoms. This is the progression of disease from compensated to decompensated cirrhosis, which is marked by jaundice, variceal bleeding, ascites, spontaneous bacterial peritonitis, and hepatic encephalopathy (HE) [53].

### 4.2. Gut Microbiome Profile in Liver Cirrhosis

The pathophysiology of liver cirrhosis is related to the gut dysbiosis that occurs in cirrhosis. A healthy liver serves as the barrier between the systemic circulation and the gut; when cirrhosis develops and progresses, this barrier is dysfunctional. There have been several studies that have shown a link between dysbiosis and disease progression in cirrhotic patients. In cirrhotic patients, there is a relative decrease in commensal autochthonous taxa such as *Lachnospiraceae*, *Ruminococcaceae*, and *Clostridia*, all of which are part of the phyla *Firmicutes*, and a decrease in *Bacteroidetes* [54,55,56]. There is also a rise in pathogenic bacteria, such as *Enterobacteriaceae*, *Veillonellaceae*, and *Streptococcaceae* [54,57,58]. Chen et al., evaluated the fecal microbiome composition of 36 patients with liver cirrhosis and 24 healthy controls, and noted that the increase in *Streptococcaceae*, a pathogenic bacterium, and the decrease in *Lachnospiraceae*, a beneficial bacterium, were associated with a higher Child-Turcotte-Pugh score, and thus worse disease prognosis [54]. Various other studies that have reported similar results from samples of stool, saliva, and intestinal mucosa are summarized in Table 2 below.

Autochthonous commensal bacteria are associated with the production of SCFAs, products of secondary bile acids from primary bile acids. Pathogenic bacteria are linked with the production of LPS and decreased SCFAs [59]. The gut microbiota is responsible for 7⍺-hydroxylation of primary bile acids into secondary bile acids [60]. Kakiyama et al., found that compared to controls, patients with cirrhosis had decreased amounts of *Lachnospiraceae*, *Ruminococcaceae*, and *Blautia*, all bacteria responsible for 7⍺-hydroxylation [61]. This decrease in bile acids is also thought to contribute to the invasion of the gut by oral commensal bacteria. *Streptococcus salivarius*, an oral commensal, was found to be increased in the gut microbiome in patients with cirrhosis who had mild HE [57]. Oral commensal bacteria of the *Veillonella* species were shown to be increased in the duodenal mucosa of patients with cirrhosis [62]. Moreover, these bacteria are urease-producing organisms. Thus, it is possible that the translocation of these bacteria to the gut contributes to the endotoxemia that is seen in patients with cirrhosis [57,58,62].

The rise in pathogenic bacteria leads to the complications of decompensated cirrhosis. The major complication of decompensated disease is HE. Ammonia is produced from bacterial metabolism of urea and proteins, nitrogenous products in the diet, and from the deamination of glutamine. Normally, the liver converts all this ammonia to urea, but in cirrhosis, the liver is unable to do this. Thus, ammonia accumulates in the blood and crosses the blood–brain barrier. The high levels of ammonia, combined with the pro-inflammatory cytokines that are produced in response to gut dysbiosis, lead to cerebral edema [63]. Certain bacteria have been linked with impaired cognition and inflammation in patients with HE. Bajaj et al., analyzed stool samples of cirrhotic patients with and without HE, and found that *Veillonellaceae*, *Fusobacteriaceae*, and *Enterobacteriaceae* were associated with worsening inflammation. They also noted that increased certain bacterial families, such as *Alcaligeneceae* and *Porphyromonadaceae*, were associated with higher levels of cognitive impairment [55]. *Alcaligeneceae* can degrade urea to ammonia and are a cause of opportunistic infections. This can explain the poor cognition in patients with HE. *Enterobacteriaceae* also have urease activity and can contribute to increased ammonia production. *Porphyromonadaceae* has also been linked to increased white matter interstitial edema [64].

**Table 2:** Studies characterizing the composition of the gut microbiota in liver cirrhosis.

Author Reference	Country	Study Design	Participants	Changes in the Composition of the Gut Microbiota in Liver Cirrhosis	Key Findings
[54]	China	Case-control	36 cirrhosis 24 healthy controls	↑ *Proteobacteria* ↑ *Fusobacteria* ↑ *Enterobacteriacea* ↑ *Veillonellacea* ↑ *Streptococcaceae* ↓ *Bacteroidetes* ↓ *Lachnospiraceae*	Fecal microbiome composition was altered in patients with cirrhosis compared to healthy individuals, indicating there is dysbiosis ↑ *Enterobacteriaceae* and *Streptococcaceae* may affect cirrhosis prognosis
[55]	United States	Prospective cohort study	25 cirrhosis: 17 with HE 8 without HE 10 controls	↑ *Bacteroidetes* ↑ *Veillonellaceae* in HE ↑ *Enterobacteriacea* ↑ *Alcaligeneceae* ↑ *Porphyromonadacea* ↑ *Fusobacteriaceae* ↓ *Ruminococcaceae* ↓ *Lachnospiraceae*	Dysbiosis was found in patients with HE compared to healthy individuals Certain bacterial families were associated with endotoxemia, impaired cognition, and inflammation in liver cirrhosis patients in HE
[57]	China	Case-control	26 cirrhosis patients with MHE 25 cirrhosis patients without MHE 26 healthy controls	↑ *Streptococcus salivarius* in HE ↑ *Streptococcaceae* ↑ *Veillonellaceae*	*Streptococcus salivarius* was positively correlated with ammonia accumulation in MHE patients
[61]	United States and Japan	Cross-sectional study	47 cirrhosis 14 healthy controls	↑ *Enterobacteriaceae* ↓ *Lachnospiraceae* ↓ *Ruminococcaceae* ↓ *Blautia*	↑ Pathogenic bacteria due to gut dysbiosis in cirrhotic patients altered bile acid composition
[58]	China	Case-control	98 cirrhosis 83 controls	↑ *Proteobacteria* ↑ *Veillonella* ↑ *Streptococcus* ↓ *Bacteroidetes*	In liver cirrhosis, there was an invasion of the gut by oral bacterial species
[64]	Unites States	Case-control	87 with HE 40 healthy controls	↑ *Enterobacteriaceae* ↓ *Lachnospiraceae* ↓ *Ruminococcaceae*	Specific bacterial families were associated with astrocytic and neuronal MRI changes Gut dysbiosis in cirrhosis was linked with systemic inflammation, elevated ammonia levels, and neuronal dysfunction
[62]	China	Case-control	30 cirrhosis 28 healthy controls	↑ *Veillonella* ↑ *Megasphaera* ↑ *Dialister* ↑ *Atopobium* ↑ *Prevotella* ↑ *Firmicutes*	↑ Oral bacteria in duodenal mucosal microbiota in cirrhotic patients
[65]	China	Cross-sectional study	36 cirrhosis 20 healthy controls	↑ *Firmicutes* ↓ *Bacteroidetes*	↑ Microbial dysbiosis in cirrhotic patients with Child-Pugh scores > 5 led to slower small bowel transit
[66]	Austria	Case-control	90 cirrhosis: 50 on PPI therapy 40 not on PPI therapy	↑ *Streptococcus salivarius* ↑ *Veillonella parvula*	↑ Gut dysbiosis in cirrhotic patients with long-term PPI therapy
[67]	Spain	Prospective cohort study	182 cirrhosis	↑ *Enterococcus* ↑ *Streptococcus* in ACLF ↑ *Faecalibacterium* ↑ *Ruminococcus* ↑ *Eubacterium* in decompensated patients	As cirrhosis progressed from compensated to uncompensated to ACLF, there was a linear progression in reduction in gene and metagenomic richness
[56]	Russia	Case-control	48 cirrhosis 21 healthy controls	↑ *Enterobacteriaceae* ↑ *Proteobacteria* ↑ *Lactobacillaceae* ↓ *Firmicutes* ↓ *Clostridia*	Severe dysbiosis was an independent risk factor for death Levels of *Clostridia* and *Bacilli* determined death within a year Levels of *Proteobacteria* and *Enterobacteriaceae* determined the long-term prognosis (death over the subsequent three years)

ACLF, acute-on-chronic liver failure; HE, hepatic encephalopathy; MHE, minimal hepatic encephalopathy; PPI, proton pump inhibitors; ↑, increase; and ↓, decrease.

## 5. Gut Microbiota: Link with Hepatocellular Carcinoma

### 5.1. Epidemiology, Clinical Manifestations, and Pathophysiology

HCC is one of the most common cancers associated with cancer-related mortality worldwide [68]. It is the ninth leading cause of cancer deaths in the United States. Despite advanced practices, screening and prevention modalities, and new therapies, it continues to be the most common primary liver malignancy. Studies have shown that HCC occurs 2.4 times more in men than in women. The incidence of liver cancer has increased from 1.6 to 4.6 per 100,000 persons among American Indigenous peoples and Alaskan Natives followed by Blacks, Whites, and Hispanics [69]. Hepatitis B (HBV), Hepatitis C (HCV), NAFLD, and NASH are linked with an increased risk of developing HCC. Other risk factors include advanced age, obesity, diabetes, family history, dietary aflatoxins exposure, alcohol, and genetic variations. Among these, HBV is the leading risk factor universally, accounting for a minimum of 50% of cases of HCC [70]. This further explains the global epidemiology of HCC; there is increased incidence in areas with endemic HBV infection including sub-Saharan Africa and eastern Asia [70].

There are several contributing factors to the pathogenesis of HCC. It is a heterogeneous malignancy associated with exposure to repeated and prolonged cycles of hepatocyte injury. HBV, HCV, and other previously mentioned risk factors cause repeated damage to the hepatocytes contributing to the formation of liver cirrhosis. Thereafter, several events at the molecular level such as gene alterations, somatic transformations, epigenetic modifications, and pathway alterations ultimately lead to the development and origination of HCC [71].

Somatic mutations can contribute to the process of initiating HCC through telomerase promoter mutations. Prolonged exposure of the hepatocytes to injury yields excessive shortening of telomeres due to increased cell turnover [72]. Telomerase reverse transcriptase (TERT) and telomerase RNA component (TERC) form telomerase, an enzymatic ribonucleoprotein complex that preserves the length of telomeres. Hereditary genetic variations in the TERT and TERC genes result in decreased telomerase activity, causing increased shortening of telomeres [71]. Not only does the resultant chromosomal instability contribute to the initiation of carcinogenesis, but it also prompts liver inflammation and ultimately cirrhosis, one of the major precursors to HCC [73]. Other common mutations include TP53 pathway mutations that further endorse oncogenic pathways. They occur in about 18–50% of HCCs [71]. Genetic factors that were found to be mutated in the p53 pathway in HCC include ATM and RPS6KA3. In addition, a link was illustrated between diet exposures to AFB1, a fungal aflatoxin, with the development of this mutation. Furthermore, HBV, one of the leading risk factors for developing HCC, has also shown an increase in incidence of G/C to T/A transversion mutation following AFB1 exposure [74]. Other common mutations are β-catenin (CTNNB1) (18–40%) and AXIN1 and AXIN2 gene mutations in the Wnt/β-catenin pathway [75].

Epigenetic modifications are inherited variations of genetic material expression by changes to the DNA structure. It does not involve DNA sequence changes. In HCC, studies have shown that DNA methylation, histone modification, chromatin remodeling, and microRNA expression are all epigenetic means of inducing carcinogenesis. For instance, a study demonstrated that methylation of 15 genes occurred in HCV-related HCC, in which the genes were part of the cancer-related RAS/RAF/ERK and Wnt/β-catenin pathways [76]. Consequently, these findings suggest the increased correlation between etiology and molecular variations as DNA methylation was linked with HBV and HCV-related HCC [77].

### 5.2. Gut Microbiome Profile in HCC

Alterations of the gut microbiome play an essential role in hepatocarcinogenesis, as demonstrated by multiple experimental human and animal studies, highlighted in Table 3 [68]. For instance, the mouse model of NASH-HCC induced by a streptozotocin-high-fat diet showed increased levels of *Firmicutes* and *Actinobacteria* and decreased *Bacteroidetes* and *Proteobacteria* species and hence an overall increase in the *Firmicutes*/*Bacteroidetes* ratio [78]. Moreover, correlation analysis between the gut microorganisms and the LPS levels demonstrated an association of LPS with the pathophysiological features associated with HCC development. There was an increase in *Atopobium*, *Bacteroides*, *Clostridium*, and *Desulfovibrio*, which showed a positive correlation with LPS. However, there was also a decrease in microbiota that negatively correlated with LPS, which may suggest potential protective and advantageous roles of the bacteria [78]. 

Grat et al., detected an elevation in *Escherichia coli* (*E. coli*) in a group of 15 patients with HCC and liver cirrhosis [79]. Similar findings were observed by Zhang et al., after DEN-induced HCC in mice, where there was a noticeable elevation in *E. coli* growth [80]. In addition, administering penicillin resulted in further gut dysbiosis and decreased *Bifidobacterium* and *Lactobacillus* species and increased LPS levels. However, probiotics instigated a decrease in *E. coli* and LPS levels. Ni et al., formed a broad index to calculate the degree of dysbiosis in primary HCC and found elevated levels of pro-inflammatory bacteria with *Proteobacteria* phyla such as *Enterobacter* and *Haemophilus* [81]. Moreover, Liu et al., also found similar findings of elevated levels of pro-inflammatory bacteria such as *Enterococcus*, *Escherichia*, and *Shigella* and reduced levels of *Faecalibacterium*, *Ruminococccus*, and *Ruminoclostridium* in non-hepatitis B and non-hepatitis C patients with HCC [82]. This group had fewer anti-inflammatory bacteria and more pro-inflammatory bacteria, which could possibly be due to their increased alcohol consumption: alcohol promotes inflammation and contributes even further to gut dysbiosis. 

Ponziani et al., noted a rise in *Ruminococcaceae* and *Bacteroides* and a decrease in *Bifidobacterium* in patients with HCC when comparing their gut microbiota in patients with cirrhosis [83]. Zheng et al.’s findings showed a decrease in butyrate-producing bacteria including *Clostridium*, *Ruminococcus*, and *Coprococcus* [84]. However, there was an increase in LPS-producing bacteria including *Neisseria*, *Enterobacteriaceae*, and *Veillonella* among patients with cirrhosis and HCC, consistent with previously mentioned studies. In conclusion, all the above findings confirm the role that gut dysbiosis maintains in in the pathogenesis and progression of HCC.

Moreover, other studies observed a relationship between gut bacteria and its products’ contribution to liver malignancy. For instance, the conversion of primary bile acids into secondary bile acids in the liver has shown to contribute to HCC pathogenesis. In an animal mice study, it was shown that secondary bile acids production and buildup in the liver were facilitated by the gut microbiota, and it may contribute to liver carcinogenesis via activation of the mTOR pathway in hepatocytes [85]. In the gut, the conversion of primary bile acids to secondary bile acids is facilitated by *Clostridium* species [86]. Secondary bile acid generation requires deconjugation of bile acids, which is facilitated by *Bacteroides* species. Therefore, an increase in these bacterial species may correlate with increased secondary bile acids accumulation and hence HCC development [87].

Studies also suggest that our current knowledge regarding gut microbiota and its alterations in HCC can potentially be utilized and allow for gut microbiota to be used as a biomarker to diagnose HCC promptly, especially considering its precision, efficiency, and non-invasiveness [80]. Future manipulation of gut microbiota through probiotics, antibiotics, or other interventions can potentially be an approach for HCC prevention. 

**Table 3:** Studies characterizing the composition of the gut microbiota in hepatocellular carcinoma.

Author Reference	Country	Study Design	Participants	Changes in the Composition of Gut Microbiota in HCC	Key Findings
Human Studies					
[79]	Poland	Cross-sectional	15 HCC 5 without HCC All participants had cirrhosis and underwent liver transplantation.	↑ *Escherichia coli* ↑ *Enterobacteriaceae* ↑ *Enterococcus* ↑ *Lactobacillus* ↑ H_2_O_2_-producing *Lactobacillus* species	↑ Fecal counts of *E coli* were noted in the cirrhotic-HCC group, demonstrating its role in HCC development
[88]	Australia	Cohort study; metagenomics and metabolomics analysis	32 NAFLD-HCC 28 NAFLD-cirrhosis 30 non-NAFLD controls	↑ *Proteobacteria* ↑ *Enterobacteriaceae* ↑ *Bacteroides xylanisolvens* ↑ *B. caecimuris* ↑ *Ruminococcus gnavus* ↑ *Clostridium bolteae* ↑ *Veillonella parvula* ↑ *Bacteroides caecimuris* ↑ *Veillonella parvula* ↑ *Clostridium bolteae* ↑ *Ruminococcus gnavus* ↓ *Oscillospiraceae* ↓ *Erysipelotrichaceae* ↓ *Eubacteriaceae*	↑ *B. caecimuris* and *Veillonella parvula* distinguished NAFLD-HCC from NAFLD-cirrhosis and non-NAFLD controls ↓ Gut microbial α-diversity ↑ SCFAs serum levels in NAFLD-HCC compared to NAFLD-cirrhosis and non-NAFLD control Gut microbiota in NAFLD-HCC microbiota contribute to immunosuppression
[89]	China	Cohort	75 with early HCC 40 liver cirrhosis 75 healthy controls	↑ *Actinobacteria* ↑ *Gemmiger* ↑ *Parabacteroides* ↑ *Paraprevotella* ↑ *Klebsiella* ↑ *Haemophilus* ↓ *Verrucomicrobia* ↓ *Alistipes* ↓ *Phascolarctobacterium* ↓ *Ruminococcus* ↓ *Oscillibacter* ↓ *Faecalibacterium* ↓ *Clostridium IV* ↓ *Coprococcus*	↓ Butyrate-producing bacteria ↑ LPS-producing bacteria in early HCC versus healthy controls
[82]	China	Case-control	57 HCC (35 with HBV related HCC, 22 with non-HBV non-HCV related HCC) 33 healthy controls	↑ *Bifidobacterium* ↑ *Lactobacillus* ↓ *Proteobacteria* ↓ *Firmicutes*	↓ Anti-inflammatory and ↑ pro-inflammatory bacteria in non-HBC non-HCV related HCC patients which correlated with their increased alcohol consumption
[81]	China	Case-control	68 with primary HCC: (23 Stage I, 13 Stage II, 30 Stage III, 2 Stage IV) 18 healthy controls	↑ Dysbiosis index *Proteobacteria* (*Enterobacter*, *Haemophilus*) ↑ *Desulfococcus* ↑ *Prevotella* ↑ *Veillonella* ↓ *Cetobacterium*	↑ Dysbiosis index in patients with primary HCC compared with healthy controls
[83]	Italy	Cohort	21 with NAFLD-related cirrhosis with HCC 20 NAFLD-related cirrhosis without HCC 20 healthy controls	↑ *Bacteroides* ↑ *Ruminococcaceae* ↓ *Bifidobacterium*	↑ Fecal calprotectin in HCC patients, which explains increased inflammation
[90]	Argentina	Case-control	407 Cirrhosis: 25 with HCC, 25 w/o HCC 25 healthy controls	↑ *Erysipelotrichaceae* ↑ *Odoribacter* ↑ *Butyricimonas* ↓ *Leuconostocaceae* ↓ *Fusobacterium* ↓ *Lachnospiraceae*	↓ *Prevotella* in cirrhotic patients with HCC, which is associated with the activation of several inflammatory pathways such as the NLR signalling pathways
[91]	China	Case-control	24 PLC 24 cirrhosis 23 healthy controls	↑ *Enterobacter ludwigii* ↑ *Enterococcaceae* ↑ *Lactobacillales* ↑ *Bacilli* ↑ *Gammaproteobacteria* ↑ *Veillonella* ↓ diversity of *Firmicutes* ↓ *Clostridia* ↓ *Subdoligranulum*	*Veillonella* positively correlated with AFP *Subdoligranulum* negatively correlated with AFP *Subdoligranulum* contains SCFA-producing lineages
[84]	China	Case-control	24 hepatitis 24 cirrhosis 75 HCC (35 with HBV, 25 with HCV, 15 with ALD) 20 healthy controls	↑ *Neisseria* ↑ *Enterobacteriaceae* ↑ *Veillonella* ↑ *Limnobacter* ↓ *Enterococcus* ↓ *Phyllobacterium* ↓ *Clostridium* ↓ *Ruminococcus* ↓ Coprococcus	↑ LPS by harmful bacteria generated liver inflammatory reactions through TLR4
**Animal experimental model studies**					
[78]	Japan	Mice	24 STZ-HFD (streptozocin-high-fat diet)-induced 24 controls	↑ *Bacteroides* ↑ *Bacteroides vulgatus* ↑ *Bacteroides uniformis* ↑ *Clostridium* ↑ *Clostridium xylanolyticum* ↑ *Clostridium fusiformis* ↑ *Roseburia* ↑ *Allobaculum* sp. *id4* ↑ *Subdoligranulum* ↑ *Anaerotruncus* ↑ *Oscillibacter* ↑ *Xylanibacter* ↑ *Mucispirillum schaedleri* ↑ *Pseudobutyrivibrio* ↑ *Desulfovibrio* ↑ *Dehalobacterium* ↑ *Oscillospira* ↑ *Sarcina* ↑ *Atopobium* ↑ *Peptococcus* ↓ *Parasutterella* ↓ *Bacteroides acidofaciens* ↓ *Odoribacter* ↓ *Barnesiella* ↓ *Moryella* ↓ *Paraprevotella* ↓ *Lactobacillus intestinalis* ↓ *Akkermansia*	*Clostridium*, *Bacteroides*, and *Desulfovibrio* were involved in bile acid dysregulation; their increased levels resulted in the preservation of high concentrations of bile acids, further contributing to hepatocarcinogenesis

AFP, alpha-fetoprotein; ALD, alcohol-associated liver disease; HBV, hepatitis B virus; HCV, hepatitis C virus; HCC, hepatocellular carcinoma LPS, lipopolysaccharide; NAFLD, nonalcoholic fatty liver disease; PLC, primary liver cancer; SCFAs, short-chain fatty acids; ↑, increase; and ↓, decrease.

## 6. Gut Microbiota: Link with Autoimmune Hepatitis

### 6.1. Epidemiology, Clinical Manifestations, and Pathophysiology

AIH is an inflammatory disorder mediated by autoimmune damage to hepatocytes. Epidemiological data on AIH vary due to the rarity of the disease. Czaja et al., found disparities in the global prevalence of AIH, ranging from as high as 42.9 cases per 100,000 persons in native Alaskans to as low as 2.4 cases per 100,000 persons in children in Canada [92]. Tunio et al., performed a retrospective analysis of a population-based database and found the prevalence to be 31.2 per 100,000 in the United States [93]. Rates of AIH are higher in women than in men, with a ratio of 3.6:1 [94]. There are two peaks in the age of onset of AIH, with the first onset in children and young adults and the second age of onset between 40–70 [95].

The underlying pathogenesis of AIH is still being elucidated; however, it involves an interaction between environmental and genetic factors. A genome-wide association study conducted by Boer et al., found an association in the major histocompatibility complex region of HLA-DRB1*0301, which would be a primary susceptibility genotype, and HLA-DRB1*0401, which would be a secondary susceptibility genotype [96]. Environmental factors include exposure to viral infections such as cytomegalovirus, Epstein–Barr virus, hepatitis A, B, C, and E, or drugs such as nitrofurantoin and minocycline [97,98,99]. Environmental factors may then precipitate the development of AIH in an individual with genetic susceptibility, whereby antigen-presenting cells present self-antigens to the T-cell receptor of CD4 T-helper cells in the liver. This in turn leads to the release of cytokines, such as IL-2 as well as IFN-gamma, via Th1 cells. This results in the expression of HLA class I and class II, the production of CD8 T cells, as well as the release of TNF-α and IL-1 via macrophages. The release of other interleukins, such as IL-4, IL-10, and IL-13 mediated by Th2 cells, results in antibody-mediated cell toxicity. Finally, Th17 cells release Il-17, Il-22, and TNF-α. Treg cells control damage to hepatocytes via the production of pro-inflammatory cytokines such as Il-10 and TGF-beta, and thus, aberrations in this pathway result in increased production of cytokines [100].

Molecular mimicry may also contribute to the etiology of AIH, in which an immune response initially targeted against foreign antigens becomes directed against self-antigens [101]. Studies have shown that mice expressing P450 2D6, a self-antigen involved in the pathogenesis of AIH-type 2, infected with adenovirus exhibited autoimmune damage to hepatocytes [102,103].

### 6.2. Gut Microbiome Profile in Autoimmune Hepatitis

Alterations in the gut microbiome may contribute to the pathogenesis of AIH. There is an overall decrease in the alpha diversity of the gut microbiome and a rise in serum LPS levels in patients with AIH [104,105,106,107,108]. LPS results in the activation of toll-like receptor 4 and NF-kB pathways which decreases the expression of tight junction proteins [109]. Several studies have shown the presence of disturbed intestinal tight junctions with reduced zona occludens-1 and occludin expression [105,107,108]. Increased intestinal permeability allows for LPS translocation to the liver, resulting in cytokine production and inflammation [109]. 

There have been only a few studies published on the gut microbiome composition in patients with AIH. Wei et al., noted a decrease in obligate anaerobes, such as *Coprococcus*, *Oscillospira*, and *Ruminococcaceae*, and a rise in facultative anaerobes, such as *Streptococcus*, *Klebsiella*, and *Lactobacillus*, in the gut microbiome of patients with AIH as noted in Table 4 [105]. Lewinksy et al., also showed a decline in obligate anaerobic bacteria, such as *Faecalibacterium*, and a rise in facultative anaerobic genera *Streptococcus* and *Lactobacillus* [106]. Several obligate anaerobic bacteria produce SCFAs, which display anti-inflammatory properties via regulating T regulatory cells and providing an energy source to the epithelium within the colon [106]. Thus, a decline in obligate anaerobes may contribute to gut dysbiosis in patients with AIH. However, other studies have noted a reduction in Lactobacillus [107,108]. 

The gut microbiome can serve as non-invasive biomarkers in AIH. Of particular note is that *Veillonella*, which belongs to the phylum *Bacilotta*, was found to be increased in the gut microbiome of patients with AIH across several studies [104,105,106]. *Veillonella* showed the most significant association with AIH, correlated with increased AST levels and advanced liver inflammation in a study conducted by Wei et al., An increase in LPS in the gut microbiome of patients with AIH may be attributed to increased *Veillonella*. Several studies have shown a decrease in *Bifidobacterium*, which belongs to the phylum *Actinomycetota*, in the gut microbiome of patients with AIH [106,107,108]. Lewinksy et al., noted that a lack of *Bifidobacterium* was associated with increased disease activity and failure to achieve remission of AIH [106]. The protective effect of *Bifidobacterium* was shown in a study by Zhang et al., whereby *Bifidobacterium* animals subs. Lactose 420 increased the production of SCFAs, the presence of intestinal tight junction proteins, as well as reduced cytokines and Th17 cells [110].

**Table 4:** Studies characterizing the composition of the gut microbiota in autoimmune hepatitis.

**Author** **Reference**	**Country**	**Study Design**	**Participants**	**Changes in the Composition of Gut Microbiota in AIH**	**Key Findings**
[107]	China	Case-control	24 AIH 8 healthy controls	↓ *Bifidobacterium* ↓ *Lactobacillus* *Escherichia coli* and *Enterococcus* were unchanged	↑ Intestinal permeability and gut dysbiosis ↑ Bacterial translocation, indicated by increased LPS, was correlated with AIH disease severity
[104]	Egypt	Case-control	5 AIH 10 healthy controls	↑ *Faecalibacterium* ↑ *Blautia* ↑ *Streptococcus* ↑ *Veillonella* ↑ *Eubacterium* ↑ *Lachnospiraceae* ↑ *Butyricicoccus* ↑ *Haemophilus* ↑ *Bacteroides* ↑ *Clostridium* ↑ *Ruminococcaceae* ↓ *Prevotella* ↓ *Parabacteroides* ↓ *Dilaster*	↓ Bacterial diversity in AIH ↑ Butyrate forming bacteria (e.g., *Butyricicoccus* and *Ruminococcaceae*)
[106]	Germany	Case-control	72 AIH 95 healthy controls 99 primary biliary cholangitis 81 ulcerative colitis	↑ *Proteobacteria* ↑ *Veillonella* ↑ *Streptococcus* ↑ *Lactobacillus* ↓ *Firmicutes* in all groups ↓ *Faecalibacterium* ↓ *Bifidobacterium*	↓ *Bifidobacterium* in AIH was associated with increased disease activity and failure to achieve remission ↓ α-diversity in AIH patients vs. healthy controls
[105]	China	Cross-sectional	119 steroid-naïve AIH 132 healthy controls	↑ *Veillonella* ↑ *Streptococcus* ↑ *Klebsiella* ↑ *Lactobacillus* ↓ *Clostridiales* ↓ *Ruminococcaceae* ↓ *Rikenellaceae* ↓ *Oscillospira* ↓ *Parabacteroides* ↓ *Coprococcus*	↑ LPS biosynthesis ↓ α-diversity *Veillonella* showed a strong association with AIH and was link with ↑ AST and progression of liver inflammation *Veillonella*, *Lactobacillus*, *Oscillospira*, and *Clostridiales* have high diagnostic value in AIH
[111]	China	Case-control	37 AIH 78 healthy controls	↑ *Veillonella* ↑ *Faecalibacterium* ↑ *Akkermansia* ↑ *Klebsiella* ↑ *Enterobacteriaceae_unclassified* ↑ *Megasphaera* ↓ *Pseudobutyrivibrio* ↓ *Lachnospira* ↓ *Ruminococcaceae* ↓ *Blautia* ↓ *Erysipelotrichaceae_incertae_sedis* ↓ *Phascolarctobacterium*	A combination of *Bacteroides*, *Ruminococcaceae*, *Lachnospiraceae*, *Veillonella*, *Roseburia*, and *Ruminococcaceae* could distinguish AIH patients from healthy controls
[108]	China	Case-control	32 AIH 20 NAFLD 20 healthy controls	↑ *Escherichia coli* ↓ *Bifidobacterium* ↓ *Lactobacillus* ↓ *Bacteroides* ↓ *C. leptum*	↑ Serum LPS in comparison to NAFLD and healthy controls

AIH, autoimmune hepatitis; LPS, lipopolysaccharide; NAFLD, nonalcoholic fatty liver disease; SCFAs, short-chain fatty acids; ↑, increase; and ↓, decrease.

## 7. Gut Microbiota: Link with Other Liver Diseases

Other liver diseases such as viral hepatitis and cholangiopathies that include primary sclerosing cholangitis (PSC) and PBC have also shown an association between dysbiosis and disease progression. For example, studies suggest that changes to gut microbial species play an important part in the development of CHB [112]. A decrease in the population of *Bifidobacteria* and *Lactobacillus* levels and an increase in *Enterococcus* and *Enterobacteriaceae* have been seen in patients with CHB [113]. In addition, a reduction in *Bacteroides* was also noticed in patients with CHB, together with alterations to the structure of the gut microbial species, signifying an important consequence of CHB [112]. Even though HCV is common globally, there is not much known about the gut microbial composition of patients with HCV. In a study, it was found that genera *Prevotella* and *Faecalibacterium*, together with *Acinetobacter*, *Veillonella*, and *Phascolarctobacterium*, were considerably higher in patients with HCV as compared to healthy individuals who had increased populations of *Ruminococcus*, *Bifidobacterium*, and some *Clostridia* [114]. It was found in a cross-sectional study that patients with HCV and end-stage liver disease patients had altered gut microbial composition and lower levels of α-diversity microbiota [115]. One possible explanation for alteration is that reduced bile production due to the gut microbiome leads to an increase in pathogenic and pro-inflammatory bacterial species, possibly increasing *Enterobacteriaceae* and *Porphyromonadaceae* and reducing *Firmicutes* (*Clostridium cluster XIVa*) [58].

An interesting pattern of dysbiosis and development of cholangiopathies have been shown by researchers. PSC is an extremely uncommon disease that leads to inflammation of the biliary tree and is mostly seen with IBD [116]. Several studies have investigated the changes in gut microbial compositions of patients with PSC and PSC-IBD. For example, one study showed that stool samples of patients with PSC had increased levels of *Veillonella* and a decrease in *Succinivibrio*, *Desulfovibrio*, *Phascolarctobacterium*, and *Coprococcus* as compared to healthy individuals [117]. Bajer et al., examined stool samples of patients with PSC and PSC-IBD and saw an increase in *Rothia*, *Enterococcus*, *Streptococcus*, *Clostridium*, *Veillonella*, and *Hemophilus*, and a decrease in *Coprococcus* when compared to healthy individuals [118]. One of the possible explanations of the changes in the gut microbiome is that inflammation due to PSC makes the gut barrier leaky. This, in turn, allows the gut microbiota and their products access to the liver where they exert their effects through inflammation [119]. Moreover, metabolites produced by microbial species such as bile acids and SCFAs are thought to contribute to PSC disease development [117,120,121]. Another cholangiopathy that has gained much attention is PBC, a chronic autoimmune liver disease that causes chronic cholestasis and biliary cirrhosis due to the damage produced by inflammation of the interlobar bile ducts [122]. Studies have indicated an association between changes in gut microbial population and PBC. For example, in a study performed by L.X. et al., patients with PBC had increased levels of *Veillonella*, *Bifidobacterium*, *Klebsiella*, and *Neisseria* while a decrease in *Bacteroides eggerthii*, *Hallella*, *Ruminococcus*, and *Megamonas* was seen as compared to healthy individuals [123]. In another study conducted by Tang et al., an increase in *Klebsiella*, *Lactobacillus*, *Clostridium*, *Pseudomonas*, *Hemophilus*, *Streptococcus*, *Veillonella*, and *Enterobacteriaceae* and a decrease in *Oscillospira*, *Faecalibacterium*, *Sutterella*, and *Bacteroides* was noticed [124].

Additional studies are required to further investigate the relationship between gut microbiota and patients who have PBC and PSC/PSC-IBD since it may help in developing treatments that involve altering the gut microbiome of the patients with PBC and PSC/PSC-IBD, producing therapeutic effects.

## 8. Gut Microbiota: Fungal and Viral Changes

Even though bacteria contribute to the majority of microbial genes found in the gut microbiota, fungi occupy a considerable biomass of over 100-fold that of bacteria. The fungi that most commonly dominate the healthier gut are *Saccharomyces*, *Malassezia*, and *Candida* [125]. The gut virome, according to the recently established gut virome database, mainly consists of bacteriophages (97.7%), eukaryotic viruses (2.1%), and archaeal viruses (0.1%) [126,127]. De novo research is starting to shed light on the role of these organisms as a normal part of the microbiota, as well as their involvement in pathologies of the gastrointestinal system [128].

Chu et al., observed the effect of the yeast, *Candida albicans*, on different alcohol-induced liver pathologies. They concluded that this commensal yeast can prove pathogenic in ALD [129]. Candialysin, a candida exotoxin, has been noted to worsen the prognosis of such diseases by inflicting epithelial damage on the liver, exacerbating the hepatocyte damage caused by the disease and further increasing the mortality of ALD [129,130]. In addition, Jiang et al., noted decreased bacterial and fungal diversity and increased viral diversity in fecal matter in patients with ALD [125]. There was also an increased abundance of *Candida* spp. and decreased *penicillium* and *Saccharomyces* in patients with alcoholic hepatitis compared controls [125]. Regarding changes in gut virome, Gao et al., documented increased *Escherichia*, *Enterobacteria*, and *Enterococcus* phages in fecal samples of alcoholic hepatitis patients when compared to control [126]. This further supports the conclusion that fungal and viral microorganisms play an important role in the normal microbiota as well as intestinal and extra-intestinal pathologies.

The most studied change in the gut mycobiota in NAFLD, according to You et al., are changes in the fungi *Saccharomyces boulardii*. Commensally, and in a healthy gut, this fungus carries out multiple functions, most notably regulating intestinal flora and neutralizing bacterial toxins [131]. *S. boulardii* has been shown to be decreased during NAFLD. You et al., identified an ameliorating effect of *S. boulardii* (through controlling the environment) when introduced to mice with hepatic steatosis, suggesting a strong causative correlation between *S. boulardii* and NAFLD [132]. Regarding the gut virome, Gao et al., noted a decrease in phage diversity in patients with NAFLD and were able to correlate certain phages with the severity of the disease [127]. For example, they noted a negative correlation between *Lactococcus* and *Leuconostoc* phages and the level of liver fibrosis in NAFLD and positive correlation between the abundance of Lactobacillus phages and the severity of liver fibrosis [127].

In liver cirrhosis, patients were often found to have an increased mycobiome diversity, including a well-noted abundance of *Basidiomycetes* (club fungi) and *Ascomycota* (sac fungi) [125,132]. You et al., cited an increased abundance of the sac fungi with worsening cirrhotic scarring during end-stage liver disease. Another retrospective study found that Epstein–Barr virus exacerbated fibrosis and liver damage in patients with liver cirrhosis, while streptococcus species in the gut virome played a vital role in the progression of cirrhosis and HE [126,133].

## 9. Therapeutic Gut–Microbiome Interaction

There have been several studies highlighting the use of gut microbiota-targeted therapeutic interventions. Treatments range from the use of probiotics, antibiotics, fecal microbial transplant, and liver transplant (LT). All these interventions aim to alter the gut microbiota in various liver diseases; select studies are listed in Table 5.

### 9.1. Probiotics and Prebiotics

The use of probiotics, prebiotics, and a mixture of both known as synbiotics have shown some positive outcomes in terms of treating liver diseases [134]. Probiotics can be found in fermented products such as yogurt, sauerkraut, and tempeh. On the other hand, prebiotics are mostly found in foods that are rich in fiber such as whole grains, fruits, and vegetables. When given as a treatment, prebiotics mostly consists of non-starch polysaccharides and oligosaccharides that stimulate the growth of beneficial bacteria, and probiotics are usually given as live microorganisms [135]. Probiotics modulate the gut microbiome by changing the number of bacteria and composition, decreasing gut permeability, reducing ammonia levels, and changing the immune response [136,137].

Probiotic treatment in experimental NAFLD mice showed that there was a decrease in endotoxemia, inflammatory cytokines (TNF-α, IL-6), total cholesterol, triglycerides, and lipid deposition [138,139,140]. Hsieh et al., noted a decrease in harmful microbial species such as *Clostridia* in the probiotic group [139]. The treatment also improved gut intestinal mucosal barrier [138]. However, no change was noted in the gut microbial diversity [42,141]. Bomhof and colleagues noticed that the usage of prebiotics such as fructooligosaccharides supplementation in NAFLD patients reduced steatosis and NAS in patients with NAFLD [142]. On the other hand, consumption of prebiotics and synbiotics have been correlated with a reduction in hepatic steatosis in patients with NASH [142,143,144]. 

In a randomized controlled trial, Horvath et al., found that taking a probiotic for 6 months enriched the gut microbiome in compensated cirrhosis patients and improved gut barrier function. There was an increase in *Alistipes shahii* in the probiotic group, which was correlated with increased neopterin levels, an antimicrobial molecule. Increased levels of *Syntrophococcus* and *Prevotella* were correlated with decreased zonulin, indicating decreased gut permeability [137]. 

In experimental AIH, probiotic treatment showed a reduction in serum transaminase and LPS translocation to the liver, regulation of cytokine production, an increase in SCFA production as well as strengthening of the intestinal barrier [109,110].

Probiotics have demonstrated their role in prevention of HCC development through stimulating an anti-inflammatory and anti-tumorigenic environment. Zhang et al. observed that probiotics, when given in high doses, were capable of altering the gut microbiota by causing a decrease in Gram-negative bacteria, including *E. coli*, *Atopobium cluster*, *B. fragilis*, and *Prevotella* [80]. They also resulted in lower serum levels of IL-6 and LPS, and higher IL-10 levels, hence further reducing inflammation. Li et al., also observed that probiotics contributed to preventing HCC progression in mice by increasing anti-inflammatory organisms such as *Prevotella* and *Oscillibacter* [145]. Moreover, prebiotics such as Kappaphyscus striatum found in K-carrageenan oligosaccharides were shown to increase NK cell activity and anti-tumor activity in mice with HCC [146]. It is worthwhile to have similar future studies with human subjects as this may provide a novel treatment for patients with HCC.

### 9.2. Antibiotics

Antibiotics have been used for modulating the gut microbiome in liver cirrhosis and HCC. In HCC, antibiotics have demonstrated their negative impact in the progression of HCC, by increasing Gram-negative bacteria *E. coli* and *Atopobium* and reducing beneficial bacteria such as *Bifidobacterium* and *Lactobacillus*, further promoting HCC development [80]. Hence, this negative association calls for caution when prescribing antibiotics to patients with HCC. In liver cirrhosis, rifaximin, a non-absorbable antibiotic, reduces ammonia production by altering microbial function. In minimal hepatic encephalopathy (MHE)-induced mice, rifaximin reduced microbial endotoxin production and secondary bile acids; however, it did not change microbial composition [147].

In liver cirrhosis, the liver parenchyma is transformed significantly. This affects drug metabolism since the liver is the principal site for that function [148]. Moreover, most drug metabolism reactions in the liver mainly depend on the blood flow and metabolic capacity of the liver, which is also altered in cirrhosis [148]. In patients with liver cirrhosis, some antibiotics have shown to contribute to renal failure, gastrointestinal bleeding, spontaneous bacterial peritonitis, and encephalopathy. Hence, factors to be considered when handling cirrhotic patients with infections include drugs pharmacokinetics, pharmacodynamics, hepatotoxicity, and likelihood of side effects [149]. The drug dosing should be modified depending on nutritional status, kidney function, adherence, and drug interaction. The most significant factor to consider is to diagnose an infection early and to start with an appropriate antibiotic regimen when dealing with cirrhotic patients [149].

### 9.3. Fecal Microbiota Transplantation (FMT)

In NAFLD, allogeneic FMT showed a decrease in the liver necro-inflammation and steatosis. An improvement in liver endothelial function was also noted; however, there was no change in duodenal microbial diversity after allogeneic and autologous FMT [141].

FMT has also been used as a therapeutic for liver cirrhosis, as highlighted in Table 5. Studies have also shown that FMT can be used in improving the cognitive ability in patients with HE [150]. Bajaj et al., showed the efficacy of using FMT in HE, where the donor sample had an increased abundance of *Lachnospiraceae* and *Ruminococcaceae* [151]. The FMT group also had improved cognition compared to controls. *Ruminococcaceae* levels were also associated with several favorable changes, including a decrease in IL-6 and LPS as well as a rise in butyrate and isobutyrate [150]. In addition, FMT enriched with *Lachnospiraceae* and *Ruminococcaceae* was associated with decreased alcohol cravings and alcohol use disorder events in patients with alcohol use disorder [152].

In AIH, one study showed an improvement in transaminase levels and restoration of the microbiome in FMT-treated mice, with an increase in *Bifidobacterium* and *Lactobacillus* and a reduction in *E. coli* [108].

### 9.4. Other Therapies

The major goal of treatment for cholangiopathies is to stop the disease progression [153]. Though the definitive treatment for PSC is LT, some studies have reported improvement in patients with PSC with high-dose ursodeoxycholic acid [154]. For PBC, there are only a few approved treatments such as ursodeoxycholic acid (UDCA) and obeticholic acid [155]. Moreover, the addition of fibrates to UDCA therapy to treat PBC have shown promising results. In a pilot study by Levy et al., patients with PBC were given fenofibrate daily for 48 weeks with standard dose of UDCA. A reduction in Alkaline phosphatase levels from 351U/L to 177U/L was noticed while taking fenofibrates. Upon stopping the treatment, an increase in Alkaline phosphatase levels was observed, suggesting the therapeutic benefits of fibrates for patients with PBC [156].

### 9.5. Liver Transplant

The definitive treatment for liver cirrhosis is transplant, which can help improve cognition and daily function. Bajaj et al., evaluated the effects of LT on gut dysbiosis. Post LT, there was increased microbial diversity, with an increase in beneficial, autochanthous bacteria such as *Ruminococcaceae*, *Lachnospiraceae*, and *Bacteroidetes*, and a decrease in pathogenic bacteria. However, healthy controls still had a gut microbiome that had higher proportions of beneficial bacteria, indicating that even post LT, there is residual dysbiosis that remains [157].

**Table 5:** Interventions targeting the gut microbiota in liver disease.

Author Reference	Country	Study Design	Intervention	Participants	Changes in the Composition of the Gut Microbiota	Key Findings
NAFLD						
[158]	Japan	Prospective cohort	Weight reduction	26 Pediatric NAFLD patients	Not mentioned in the study	↓ In liver stiffness and fat deposition
[138]	China	Animal experimental model (rats)	Probiotics (cholesterol-lowering probiotics and anthraquinone from *Cassia obtusifolia* L.)	30 male rats: 6 NAFLD 18 NAFLD rats received treatment 6 normal diet	↑ *Bacteroides* ↑ *Lactobacillus P* ↑ *Arabacteroides* ↓ *Oscillospira*	Probiotic use ameliorated intestinal mucosal barrier ↓ Endotoxemia and inflammatory cytokines
[139]	China	Animal experimental model (mice)	Probiotics (*Lactobacillus reuteri GMNL-263*)	12 male mice: 6 HS received treatment 6 controls	↑ *Bifidobacteria* ↑ *Lactobacilli* ↓ *Clostridia*	↓ BG levels, TNF-α and IL-6 production by adipose tissue in those taking probiotics Probiotics also modulate insulin level and can prevent type 2 diabetes
[140]	China	Animal experimental model (mice)	Probiotics	24 male mice: 8 NAFLD no treatment 8 NAFLD with treatment 8 controls	↑ *Ruminococcu* ↑ *Saccharibacteria* (*TM7 phylum*) ↓ *Verrucomicrobia* ↓ *Veillonella*	↓ TC, TG, lipid deposition and inflammation in the probiotic groups
[141]	Netherlands	Double-blind, randomized controlled	FMT (allogenic vs autologous)	21 NAFLD patients: 10 allogenic 11 autologous	Allogenic FMT: ↑ *Ruminococcus* ↑ *Eubacterium hallii* ↑ *Faecalibacterium* ↑ *Prevotella copri* Autologous FMT: ↑ *Lachnospiraceae*	Improved liver endothelial function ↓ Liver necro-inflammation and steatosis There was no change in duodenal microbial diversity in both groups
[42]	China	Randomized control trial	Probiotics	16 NASH: 7 received treatment 9 no treatment 22 healthy controls	↑ *Parabacteroide* ↑ *Allisonella* ↓ *Faecalibacterium* ↓ *Anaerosporobacter*	Bacterial biodiversity did not differ between NASH patients and controls and did not differ with probiotic treatment ↑ *Bacteroidetes* and ↓ *Firmicutes* was noted in the probiotic group
**Liver Cirrhosis**						
[159]	Czech Republic	Double-blind randomized clinical trial	Probiotics (*E. coli Nissle* strain)	39 cirrhosis patients: 17 placebo 22 treatment group	↑ *Lactobacillus species* ↑ *Bifidobacterium species* ↓ *Proteus hauseri* ↓ *Citrobacter species* ↓ *Morganella species*	Statistically significant improvement in gut microbiome in those taking the probiotic for 42 days ↓ Endotoxemia, bilirubin, and ascites
[136]	India	Double-blind, randomized, placebo-controlled clinical trial	Probiotics (VSL #3)	130 cirrhosis patients: 66 probiotic group 64 placebo group	↑ *Lactobacillus species*	↓ Hospitalization due to HE with daily intake of the probiotic for 6 months
[160]	United States	Double-blind, randomized, placebo-controlled clinical trial (phase I)	Probiotics (*Lactobacillus GG*)	30 cirrhosis patients: 14 probiotic group 16 placebo group	↑ *Firmicutes species* ↓ *Enterobacteriaceae* ↓ *Porphyromonadacea*	↓ Endotoxemia and TNF-α in patients taking probiotic for 8 weeks ↓ Dysbiosis due to decreased *Enterobacteriaceae* and increased *Firmicutes* species
[151]	United States	Randomized clinical trial	FMT	20 HE patients: 10 FMT 10 placebo	↑ *Lactobacillaceae* ↑ *Bifidobacteriaceae* ↑ *Bacteroidetes* ↑ *Firmicutes*	Reduction in hospitalizations, improved cognition, improved dysbiosis, and SCFAs in FMT group
[157]	United States	Case-control	Liver transplant	45 liver transplant patients 45 healthy controls	↑ *Ruminococcaceae* ↑ *Lachnospiraceae* ↓ *Enterobacteriaceae*	Post LT: ↓ pathogenic bacteria ↑ gut diversity and ↑ autochthonous bacteria Compared to controls, there was still residual dysbiosis
[161]	United States	Case-control	Periodontal therapy	24 cirrhosis patients, no therapy 26 cirrhosis patients, periodontal therapy 20 healthy controls, periodontal therapy	↑ *Ruminococcaceae* ↑ *Lachnospiraceae* ↓ *Enterobacteriaceae* ↓ *Porphyromonadaceae* ↓ *Streptococcaceae* (oral origin)	↓ dysbiosis and endotoxemia with periodontal therapy for 30 days, especially in those who had HE
[137]	Austria	Randomized clinical trial	Probiotics (multispecies strain)	26 cirrhosis patients on probiotic therapy 32 cirrhosis patients on placebo	↑ *Lactobacillus* (*brevis*, *salivarius*, *lactis*) ↑ *Faecalibacterium prausnitzii* ↑ *Syntrophococcus sucromutans* ↑ *Alistipes shahii* ↑ *Bacteroides vulgatus* ↑ *Prevotella*	Probiotic therapy for 6 months enriched the gut microbiome in compensated cirrhosis patients and improved gut barrier function Changes seen were transient
**HCC**						
[80]	China	Animal experimental model (rats)	Probiotics (VSL #3) Antibiotics (penicillin)	13 DEN-induced HCC mice: 7 probiotics 6 controls Penicillin group Dextran Sulfate sodium (DSS) group DEN + DSS + Penicillin group	↓ *Escherichia coli* ↓ *Atopobium cluster* ↓ *B. fragilis* ↓ *Prevotella* ↑ *Escherichia coli* ↑ *Atopobium* ↓ *Bifidobacterium* ↓ *Lactobacillus*	High-dose probiotic administration into DEN-induced HCC mice showed a restoration of gut homeostasis and inhibition of DEN-induced hepatocarcinogenesis There was an association between increased gut dysbiosis, inflammation, intestinal mucosa damage in the penicillin groups and the increased cell proliferation, hence demonstrating the contribution of antibiotics to hepatocarcinogensis
[145]	China	Animal experimental model (mice)	Probiotics (Prohep: *Lactobacillus rhamnosus GG* (LGG), viable *Escherichia coli Nissle 1917* (EcN), and heat-inactivated VSL#3)	8 probiotics 8 cisplatin 8 control	↑ *Alistipes* ↑ *Butyricimonas* ↑ *Mucispirillum* ↑ *Oscillibacter* ↑ *Parabacteroides* ↑ *Paraprevotella* ↑ *Prevotella* ↑ *Bacteroidetes* ↓ *Firmicutes* ↓ *Proteobacteria*	In the probiotics group: ↑ anti-inflammatory bacteria ↓ Th17-inducing bacteria and segmented filamentous bacteria which are pro-inflammatory This stayed the same in control group
**AIH**						
[109]	China	Animal experimental model (mice)	Probiotics (*Bifidobacterium* and *Lactobacillus*)	16 experimental AIH mice, no treatment 13 experimental AIH mice, probiotics 13 experimental AIH mice, dexamethasone 16 controls	↑ *Bacteroidetes* ↑ *Bifidobacterium* ↑ *Bacteroides* ↑ *Clostridium* ↑ *Ruminococcus* ↑ *Anaerostipes* ↑ *Blautia* ↓ *Firmicutes* ↓ *Faecalibacterium* ↓ *Helicobacter* ↓ *Staphylococcus*	Probiotics group: ↑ Treg differentiation ↑ SCFAs ↓ infiltration of inflammatory cells in the liver ↓ ALT, AST ↓ Th1, Th17 cells (-) LPS translocation to the liver (-) activation of the TLR/NF-kB pathway
[110]	China	Animal experimental model (mice)	Probiotics (*Bifidobacterium* animalis spp. *Lactis* *420*)	6 experimental AIH mice, no treatment 6 experimental AIH mice, probiotic 6 controls	↑ *Lactobacillus* ↑ *Alistipes* ↑ *Rikenella* ↑ *Clostridia* ↓ *Bacteroides* ↓ *Ruminococcus*	Probiotics reduced liver injury and improved immune homeostasis via: Upregulation of tight junction proteins ↓ Serum endotoxin levels ↑ Fecal SCFAs ↑ α-diversity Regulation of pro-inflammatory cytokines (-) RIP3-MLKL signalling pathway of liver macrophages
[108]	China	Animal experimental model (mice)	FMT	Antibiotic-induced gut dysbiosis AIH group, FMT therapy AIH group, FMT therapy Control group	↑ *Bifidobacterium* ↑ *Lactobacillus* ↓ *Escherichia coli*	↓ AST, ALT and serum IgG, regulation of TFR/TFH immune imbalance and restoration of microbiome in both treatment groups, thus slowing AIH progression in mice

AIH, autoimmune hepatitis; BG, blood glucose; FMT, fecal microbiota transplant; HE, hepatic encephalopathy; LT, liver transplant; N/A, not applicable; SCFAs, short-chain fatty acids; TC, total cholesterol; TG, triglyceride; TNF-α, tumor necrosis factor alpha; ↑, increase; ↓, decrease; and (-), inhibited.

## 10. Conclusions

The gut microbiota plays a significant role in the development and progression of liver diseases. Research suggests that a disturbance to the gut microbiome leads to hepatic steatosis, liver inflammation, HE, and fibrosis. These pathological processes favor the development and progression of liver diseases that include NAFLD, NASH, cirrhosis, HCC, AIH viral hepatitis, and cholangiopathies. Current studies show an association between changes in different microbiota strains and liver diseases. There has been some success with treatments that involve manipulating microbial populations through treatment with prebiotics, probiotics, antibiotics, and FMT. However, further studies are needed to provide convincing evidence on the interplay between liver diseases and changes to microbial composition and their metabolites. The use of advanced sequencing and cultural techniques may help provide more information. Secondly, it is also important to explore the specific microbial strains involved in each liver disease. Knowledge of both harmful and defensive microbial strains is essential to produce effective treatments. Thirdly, clinical trials in different settings are needed to manipulate specific microbial strains using prebiotics, probiotics, antibiotics, and FMT in populations of patients with liver disease in order to better establish a causal relationship between changes in gut microbiota and liver diseases. In conclusion, a better understanding of changes to gut flora using metagenomics and metabolomics studies can allow us to produce promising treatments for liver diseases that involve manipulating the gut microbial composition. It is possible to even have personalized treatments for individual patients based on the descriptive data of their gut microbiome acquired by analytical tools.

## Data Availability

Not applicable.

## Abbreviations

The following abbreviations are used in this manuscript:

AIH	Autoimmune hepatitis
ALD	Alcohol-associated liver disease
CHB	Chronic hepatitis B
*E. coli*	*Escherichia coli*
FMT	Fecal microbiota transplant
HBV	Hepatitis B virus
HCC	Hepatocellular carcinoma
HCV	Hepatitis C virus
HE	Hepatic encephalopathy
IBD	Inflammatory bowel disease
LPS	Lipopolysaccharide
LT	Liver transplant
NAFLD	Nonalcoholic fatty liver disease
NASH	Nonalcoholic steatohepatitis
PBC	Primary biliary cholangitis
PSC	Primary sclerosing cholangitis
PSC-IBD	Primary sclerosing cholangitis-inflammatory bowel disease
shortSCFAs	Short-chain fatty acids
TNF-α	Tumor necrosis factor alpha
